# Biological autoluminescence for assessing oxidative processes in yeast cell cultures

**DOI:** 10.1038/s41598-021-89753-9

**Published:** 2021-05-25

**Authors:** Petra Vahalová, Kateřina Červinková, Michal Cifra

**Affiliations:** Institute of Photonics and Electronics of the Czech Academy of Sciences, Prague, Czechia

**Keywords:** Biophotonics, Biological physics, Biomedical engineering, Biophysics, Biotechnology

## Abstract

Nowadays, modern medicine is looking for new, more gentle, and more efficient diagnostic methods. A pathological state of an organism is often closely connected with increased amount of reactive oxygen species. They can react with biomolecules and subsequent reactions can lead to very low endogenous light emission (biological autoluminescence—BAL). This phenomenon can be potentially used as a non-invasive and low-operational-cost tool for monitoring oxidative stress during diseases. To contribute to the understanding of the parameters affecting BAL, we analyzed the BAL from yeast *Saccharomyces cerevisiae* as a representative eukaryotic organism. The relationship between the BAL intensity and the amount of reactive oxygen species that originates as a result of the Fenton reaction as well as correlation between spontaneous BAL and selected physical and chemical parameters (pH, oxygen partial pressure, and cell concentration) during cell growth were established. Our results contribute to real-time non-invasive methodologies for monitoring oxidative processes in biomedicine and biotechnology.

## Introduction

One of the main missions of modern society is to prevent, manage, and cure diseases. A key requirement to achieve this is the development of effective and ideally non-invasive diagnostic methods. New gentle diagnostic methods will help to overcome limitations of current methods, which can be painful for the patient and destructive for the analyzed biosamples. In addition to human diseases, the development of new diagnostic methods is also important for monitoring diseases of animals or plants, which have a strong impact on agriculture and the food industry. In this work, we contribute to the development of a non-invasive biophotonic method for real-time monitoring of oxidative processes in cell cultures as model organisms.

Multiple diseases, including those that are the leading causes of deaths worldwide, such as cancer^1^, neurodegenerative^2^, and cardiovascular^3,4^ diseases, are closely related to an increased amount of reactive oxygen species (ROS) in affected body parts. In living organisms, incomplete reduction of ${\text {O}}_2$ during respiration is considered to be a typical source of ROS. However, ROS are also naturally produced during fatty acid metabolism in peroxysomes and by NADPH oxidases in the membrane of macrophages^5^. Non-mitochondrial ROS sources, originating from proteins encoded by NADPH oxidase orthologs, have also been recently discovered in single cell organisms, such as yeast^6^. The level of ROS in an organism can also be increased by ionizing radiation or chemicals^7^. To maintain redox homeostasis, which is necessary for proper functioning of an organism, organisms have developed various defense mechanisms to eliminate undesirable ROS or prevent the damage caused by them. Endogenous antioxidants include antioxidant enzymes (e.g., catalase, superoxide dismutase, or glutathione reductase), metal binding proteins (such as ferritin binding iron or ceruloplasmin binding copper), and several other compounds (e.g., lipoic acid, NAD(P)H, or thioredoxin)^8^. Water-soluble ascorbic acid or lipid-soluble tocopherol are examples of antioxidants usually supplied by nutrients.

Interactions of ROS with biomolecules lead to a cascade of reactions. ROS are initiators of these reactions but also their products. We can distinguish radicals with unpaired electron (e.g., superoxide anion ${\text {O}}_2^{- \cdot}$, peroxynitrite ${\text {ONOO}}^{-\cdot }$, hydroperoxyl ${\text {HO}}_2^{\cdot }$, hydroxyl ${\text {HO}}^{\cdot }$, peroxyl ${\text {ROO}}^{\cdot }$, alkoxyl ${\text {RO}}^{\cdot }$, or alkyl ${\text {R}}^{\cdot }$ radicals) and less reactive two-electron ROS (e.g., hydrogen peroxide ${\text {H}}_2 {\text {O}}_2$, organic hydroperoxides ROOH, or singlet oxygen $^1 {\text {O}}_2$) or if they are organic or not^7^. However, these oxidants can convert among themselves under particular circumstances and reaction conditions. The probable mechanism from the reaction of ROS with biological matter to final photon emission (BAL) is briefly described below.

**Figure 1:**
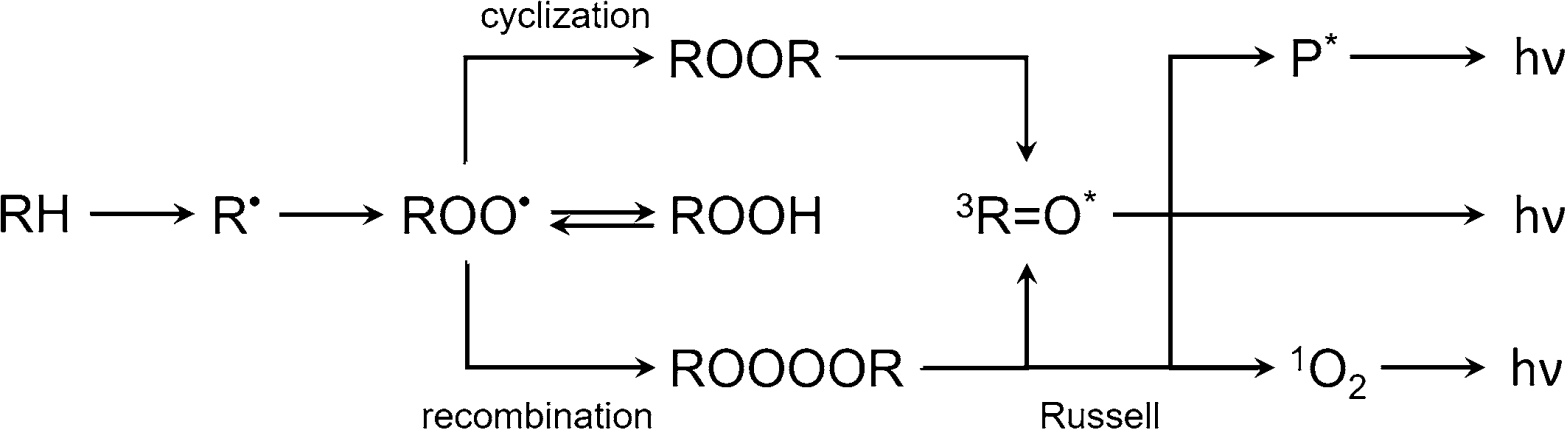
A simplified scheme of reactions of ROS with biological matter and subsequent possible reactions leading to photon emission based on earlier work^9^. Initially, the reaction of ROS with a biomolecule RH leads to the formation of various radicals, which in turn lead to the formation of unstable high-energetic intermediates (ROOR, ROOOOR). Decomposition of these energetic intermediates leads to the formation of electron excited species and subsequent photon emission h$\nu$. See details in the text.

Highly reactive non-organic radicals (mainly ${\text {HO}}^{\cdot }$ or ${\text {HO}}_2^{\cdot }$) adopt an electron from organic matter RH resulting in the formation of an organic radical ${\text {R}}^{\cdot }$^9^. In the presence of molecular oxygen, peroxyl ${\text {ROO}}^{\cdot }$ can be created and subsequently remove the hydrogen from another RH, leading to the formation of ROOH and the restoration of an alkyl ${\text {R}}^{\cdot }$ radical. Cyclization or recombination of ${\text {ROO}}^{\cdot }$ results in the creation of unstable high-energy intermediates, dioxetanes ROOR^10^ and tetroxides ROOOOR^11^, respectively. 1,2-dioxetane decomposes to triplet excited $^3 \hbox {R} {=} {\hbox {O}}^*$ and ground R=O carbonyls, and tetroxide, either to triplet excited carbonyl $^3\hbox {R} {=} {\hbox {O}}^*$, molecular oxygen, and organic hydroxide ROH or to ground-state carbonyl R=O, singlet oxygen $^1{\text {O}}_2$, and ROH^9^. If a chromophore or molecular oxygen are in proximity of triplet excited carbonyls $^3 \hbox {R}= {\hbox {O}}^*$, energy transfer can occur and lead to the formation of a triplet $^3 {\hbox {P}}^*$ or singlet $^1 {\hbox {P}}^*$ excited chromophore and singlet oxygen $^1 {\text {O}}_2$, respectively. Singlet oxygen can also be the primary product of decomposition of unstable high-energy intermediates. Redundant energy of excited species can be released in the form of heat or as light emission^12^. Triplet excited carbonyls $^3 \hbox {R}= {\hbox {O}}^*$ emit between 350 and 550 nm, chromophores in the region 550–750 nm ($^1 {\hbox {P}}^*$) and 750–1000 nm ($^3 {\hbox {P}}^*$), depending on their type. Typical emission wavelengths for singlet oxygen $^1 {\text {O}}_2$ are 1270 nm for monomol or 634 and 703 nm for dimol photon emission. A simplified scheme of the reactions is shown in Fig. 1.

However, the probabilities of the reactions leading to the formation of excited species and subsequent radiative de-excitation are very low; therefore, the intensity of photon emission is also very low^13^. From tens to several hundreds of photons $\, {\hbox {s}}^{-1} \,\, {\hbox {cm}}^2$ is the range of intensities typical for spontaneous photon emission of a sample^12^. The intensity can increase by 2–3 orders of magnitude in the case of artificially induced photon emission. On the other hand, detector efficiency, distance between the sample and detector, and additional optical effects (such as absorption or scattering in the sample) decrease the amount of photons that can be detected. Several terms are used to describe this phenomenon. Here we use biological autoluminescence (BAL) to emphasize the biological and endogenous origin of this luminescence phenomenon. Other terms used in the literature are ultra-weak photon emission, biophotons, or bio-chemiluminescence^12^.

Although the probable general biochemical and biophysical mechanisms of BAL are rather well established^13^, the origin and physiology of the processes leading to BAL are different for different organisms in different conditions^14–17^.

The amount of ROS in organisms can be affected by endogenous processes but also by the presence of external oxidants and pro-oxidants. Natural and artificial processes can lead to the generation of various types of ROS. One of the common reactions that yields highly reactive hydroxyl radicals besides other products^18^ is the Fenton reaction, which involves divalent iron (${\hbox {Fe}}^{2+}$) and hydrogen peroxide (${\text {H}}_2 {\text {O}}_2$). In organisms, these two chemicals are strictly controlled and separated. However, artificial usage of the Fenton reaction is popular in many industrial fields in which production of the highly reactive radical is desirable.

As a biological system for the analysis of ROS-BAL correlations, we chose yeast *Saccharomyces cerevisiae*, which is a widely used and well characterized model organism in molecular biology research. Furthermore, yeasts and their various strains are also essential in the food industry^19,20^ and biotechnology^21^. They are eukaryotic, which is the same as humans, animals, or plants, but single-celled organisms; thus, it is easier to evaluate the effects of various stress factors on the organism.

**Figure 2:**
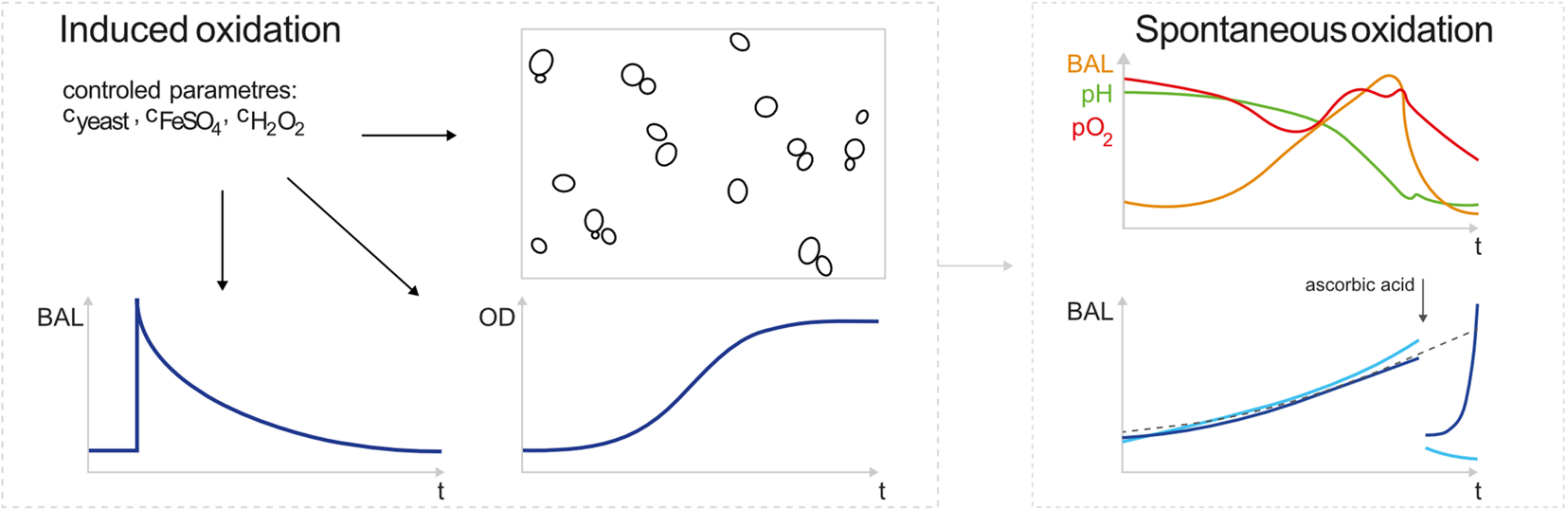
A schematic summary of the parameters and processes related to biological autoluminescence (BAL) explored in this paper. We analyzed the BAL and related parameters both under the conditions of induced and spontaneous (endogenous) oxidation in the yeast cell cultures. For induced oxidation, the amount of free radicals in a sample was regulated (increased) by the Fenton reaction. Then, the effects of various concentration of yeast and Fenton reagents (${\text {FeSO}}_4$ and ${\text {H}}_2 {\text {O}}_2$) on BAL, growth curves, and cell clustering was analyzed. The BAL due to spontaneous oxidation processes and its correlation with several selected parameters (pH, ${\text {pO}}_2$, concentration of antioxidant ascorbic acid) were established.

There have been several studies of spontaneous BAL from yeast. For example, Quickenden et al.^14,22–25^ studied the intensity and spectral distribution of spontaneous BAL in the exponential and stationary phase of yeast growth. Other researchers have used various methods to study oxidation caused by the Fenton reaction^26,27^. Ivanova et al.^28^ focused on mechanisms of chemiluminescence as a result of the Fenton reaction. To connect these topics and broaden the knowledge about them, we decided to evaluate the correlation between BAL of a living organism, yeast *Saccharomyces cerevisiae*, and artificially increased amount of ROS by the Fenton reaction. We also investigated the correlation between spontaneous BAL and selected physical and chemical parameters (pH, oxygen partial pressure, cell concentration, and concentration of the antioxidant ascorbic acid) to verify the hypothesis of ROS-BAL correlation. See Fig. 2 for the scheme of the paper.

## Results and discussion

### Biological autoluminescence enhanced by Fenton reagents

**Figure 3:**
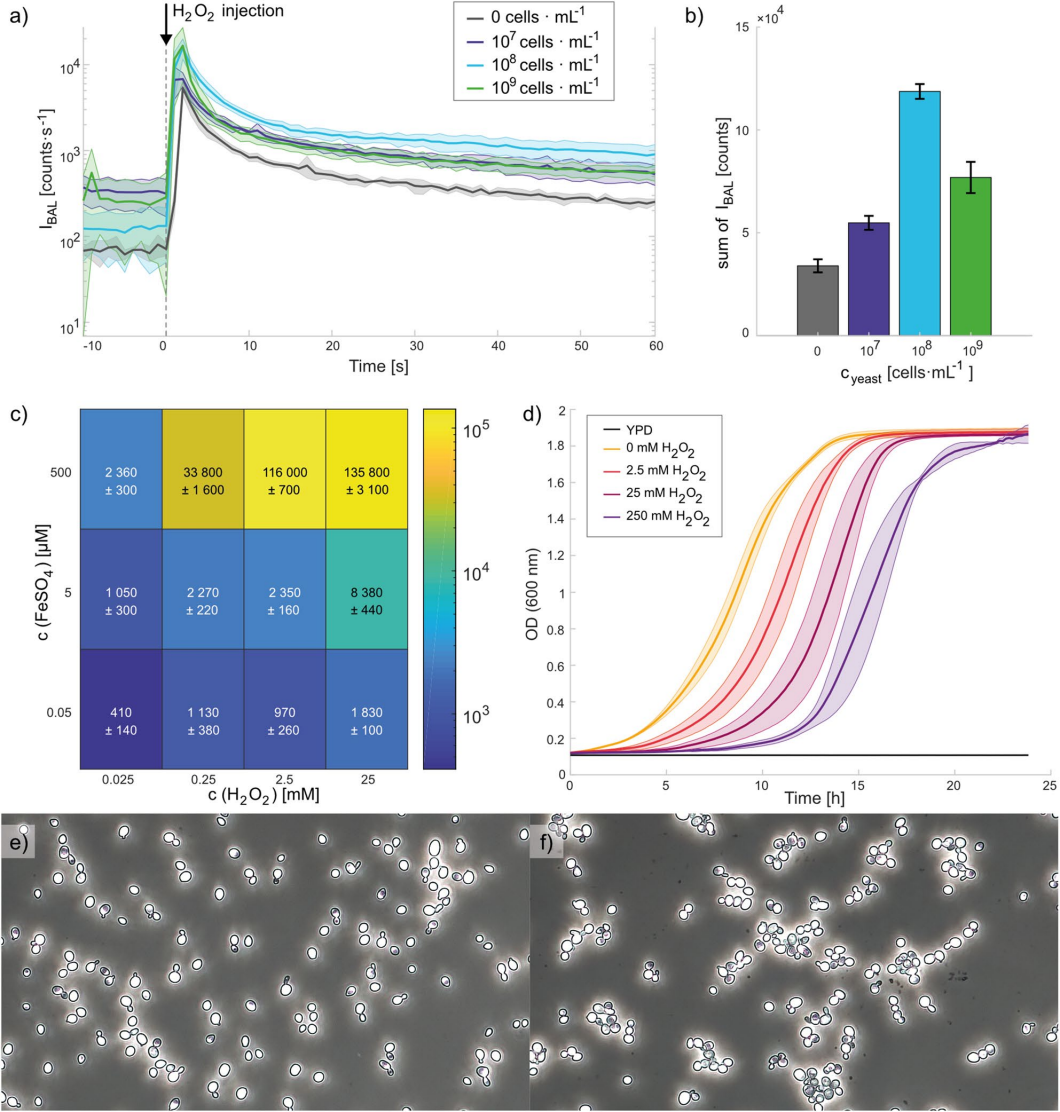
Effects of the Fenton reaction on BAL of yeast, their growth curve, and evaluation of the oxidised yeast under a microscope. First two graphs (**a,b**) show induced BAL of yeast by the Fenton reaction (0.5 mM $${\text {FeSO}}_4$$, 2.5 mM $${\text {H}}_2$$$${\text {O}}_2$$) for different concentrations of yeast ($$10^7$$–$$10^9 \, \hbox {cells} \,\, {\hbox {mL}}^{-1}$$). Part **c)** displays induced yeast BAL by the Fenton reaction for various concentrations of Fenton reagents (50 nM–0.5 mM $${\text {FeSO}}_4$$, $$25\, \upmu \hbox {M}$$–$$25\, \hbox {mM} \, {\text {H}}_2 {\text {O}}_2$$) and a fixed yeast concentration ($$10^{8} \, \hbox {cells} \,\, {\hbox {mL}}^{-1}$$). (**a**) BAL kinetics after the injection of hydrogen peroxide into the sample after 10 seconds of background measurement (t = 0). (**b**) Bar graph and (**c**) heat map of the sum of the BAL intensities during the first 60 s after injection of $${\text {H}}_2 {\text {O}}_2$$ with a subtracted background (the previous 10 s before the injection of $${\text {H}}_2 {\text {O}}_2$$). (**d**) Yeast growth curves after oxidation of cells ($$10^8 \, \hbox {cells} \,\, {\hbox {mL}}^{-1}$$) by the Fenton reaction ($$\sim$$10 min; 0.5 mM $${\text {FeSO}}_4$$, 0/2.5/25/250 mM $${\text {H}}_2 {\text {O}}_2$$) depicted by OD at 600 nm. In all cases (**a**–**d**), the average from 3 measurements and the standard deviation are shown. Microscopic pictures of a yeast sample in water (**e**) before and (**f**) after the Fenton reaction ($$\sim$$10 min; 0.5 mM $${\text {FeSO}}_4$$, 2.5 mM $${\text {H}}_2 {\text {O}}_2$$). Yeast concentrations (3–9 $$\upmu \hbox {m}$$ in a diameter) were $$10^8 \, \hbox {cells} \,\,{\hbox {mL}}^{-1}$$ before and $$3 \cdot 10^7 \, \hbox {cells} \,\, {\hbox {mL}}^{-1}$$ after oxidation.

First, we evaluated the BAL signal from yeast samples containing three different cell concentrations to determine which concentration resulted in the highest BAL intensity. The BAL intensity was observed during real-time oxidation of yeast cells with 0.5 mM ${\text {FeSO}}_4$ and 2.5 mM ${\text {H}}_2 {\text {O}}_2$ (final concentrations are always provided, unless noted otherwise). The kinetics during the first 60 s are shown in Fig. 3a. Hydrogen peroxide was injected into the sample after 10 seconds of background measurement. The sums of the BAL signal during the first 60 s after injection of ${\text {H}}_2 {\text {O}}_2$ with a subtracted background (first 10 s before the injection of ${\text {H}}_2 {\text {O}}_2$) are shown in Fig. 3b. The average from 3 independent measurements and standard deviation are displayed. We can see that the BAL signal increases together with the yeast concentration up to the limit $10^8\,\hbox {cells} \,\, {\hbox {mL}}^{-1}$. With increasing yeast concentration, the amount of biomass that ROS can react with is higher and the amount of potential sources of BAL grows. However, above $10^8\, \hbox {cells} \,\, {\hbox {mL}}^{-1}$, the samples start to become increasingly turbid and also sediment faster, making the lower layers inaccessible for oxygen and the Fenton reagents, hence decreasing the actual amount of biomass that could be directly oxidised. All these factors, we believe, lead to the lower BAL intensity for cell concentrations above $10^8\, \hbox {cells} \,\, {\hbox {mL}}^{-1}$. Therefore, for subsequent experiments we used a yeast concentration of $10^8\, \hbox {cells} \,\, {\hbox {mL}}^{-1}$, which exhibited the highest BAL intensity.

We measured the BAL response of yeast cell cultures at a concentration of $10^8\,\hbox {cells} \,\, {\hbox {mL}}^{-1}$ induced by the addition of various concentrations of the Fenton reagents ${\text {FeSO}}_4$ and ${\text {H}}_2 {\text {O}}_2$. The final concentration ranges of the reagents were 50 nM–0.5 mM ${\text {FeSO}}_4$ and 25 $\upmu$M–25 mM ${\text {H}}_2 {\text {O}}_2$. The sums of the BAL intensities during the first 60 s after injection of ${\text {H}}_2 {\text {O}}_2$ with a subtracted background (calculated from the previous 10 s before the injection of ${\text {H}}_2 {\text {O}}_2$) are shown in Fig. 3c. The given numbers (represented by colours) are the average from 3 measurements with a standard deviation. At the lowest used concentrations ($50\,\hbox {nM}\, {\text {FeSO}}_4$ and $25\,\upmu \hbox {M} \, {\text {H}}_2 {\text {O}}_2$), a signal comparable to the intensity of a control sample (only Milli-Q water without yeast) was obtained, see Fig. S1. For the measurements at higher concentrations, the BAL signals were substantially higher than those obtained with the control samples. Generally, higher Fenton reagent concentrations resulted in a higher BAL signal.

The influence of oxidation induced by the Fenton reaction (caused by the $0.5\,\hbox {mM} \, {\text {FeSO}}_4$ and 2.5 mM or $25\,\hbox {mM} \, {\text {H}}_2 {\text {O}}_2$) on a yeast cells was evaluated under a microscope. In Fig. 3e,f yeast (e) before and (f) after 10 min oxidation with 0.5 mM ${\text {FeSO}}_4$, $2.5\, \hbox {mM} \, {\text {H}}_2 {\text {O}}_2$ are shown. The yeast concentration (evaluated for cells with a diameter between 3 and 9 ${\upmu } \hbox {m}$) was $10^8\, \hbox {cells} \,\, {\hbox {mL}}^{-1}$ for control sample and before oxidation (Fig. 3e) and $3 \cdot 10^7 \, \hbox {cells} \,\, {\hbox {mL}}^{-1}$ after oxidation of the cells (Fig. 3f). For both ${\text {H}}_2 {\text {O}}_2$ concentrations, we did not observe any damaged cells. However, the yeast cells tended to cluster after oxidation. Cell aggregation could be one of the possible reasons for the lower yeast concentration that was measured in the 3–9 $\upmu \hbox {m}$ cell diameter range.

Toledano et al. claimed that the toxic concentration of hydrogen peroxide for yeast is approximately 5 mM (depending on the yeast species)^5^, [chap. 6, p.246], which is within the range of the concentrations we evaluated. However, inducing the apoptosis by external oxidants depends on the incubation time. There are also several factors that can modulate the resistance to oxidative stress. For example, yeasts have a higher resistance to oxidative stress in the stationary phase and during respiration, when their metabolic processes (oxidative phosphorylation) employ oxygen and inevitably generate ROS endogenously. Also, previous mild stress condition (including different types of stress) can lead to increased tolerance to oxidative stress. For example, the sudden transfer of yeast cells from room temperature to $30\,^\circ \hbox {C}$ and change of the medium can be experienced as stress^5^, [chap. 6]. To assess the viability of cells after the oxidation by Fenton reagents (10 min incubation; $0.5\,\hbox {mM} \, {\text {FeSO}}_4$, 2.5–250 $\hbox {mM} \, {\text {H}}_2 {\text {O}}_2$), we performed a Trypan blue assay using identical experimental conditions (yeast strain, buffers, all reagent and cells concentration, duration of oxidation treatments) in our recent work^29^. Under our experimental conditions, the cell viability remained ${>}98\,\%$ after treatment with 2.5 mM and $25~\hbox {mM} \, {\text {H}}_2 {\text {O}}_2$ and reduced to 87 % after treatment with $250~\hbox {mM} \, {\text {H}}_2 {\text {O}}_2$.

The effect of the Fenton reaction ($\approx \, 10\,\hbox {min}$ incubation; $0.5\,\hbox {mM} \, {\text {FeSO}}_4$, $2.5 \, \hbox {-} \, 250\, \hbox {mM} \, {\text {H}}_2 {\text {O}}_2$) on yeast growth was also evaluated here. As shown in Fig. 3d, the samples treated with a higher concentration of hydrogen peroxide had a longer lag phase. However, the final amount of cells (in the stationary phase) was similar for all samples. The duration of the lag phase depends on several factors such as the temperature, aeration, the conditions in a previous environment, amount of transferred biomass, or yeast health^30^, [chap. 9]. More stressed cells probably need more time to overcome unfavourable consequences and to prepare for cell division.

### Spontaneous biological autoluminescence

**Figure 4:**
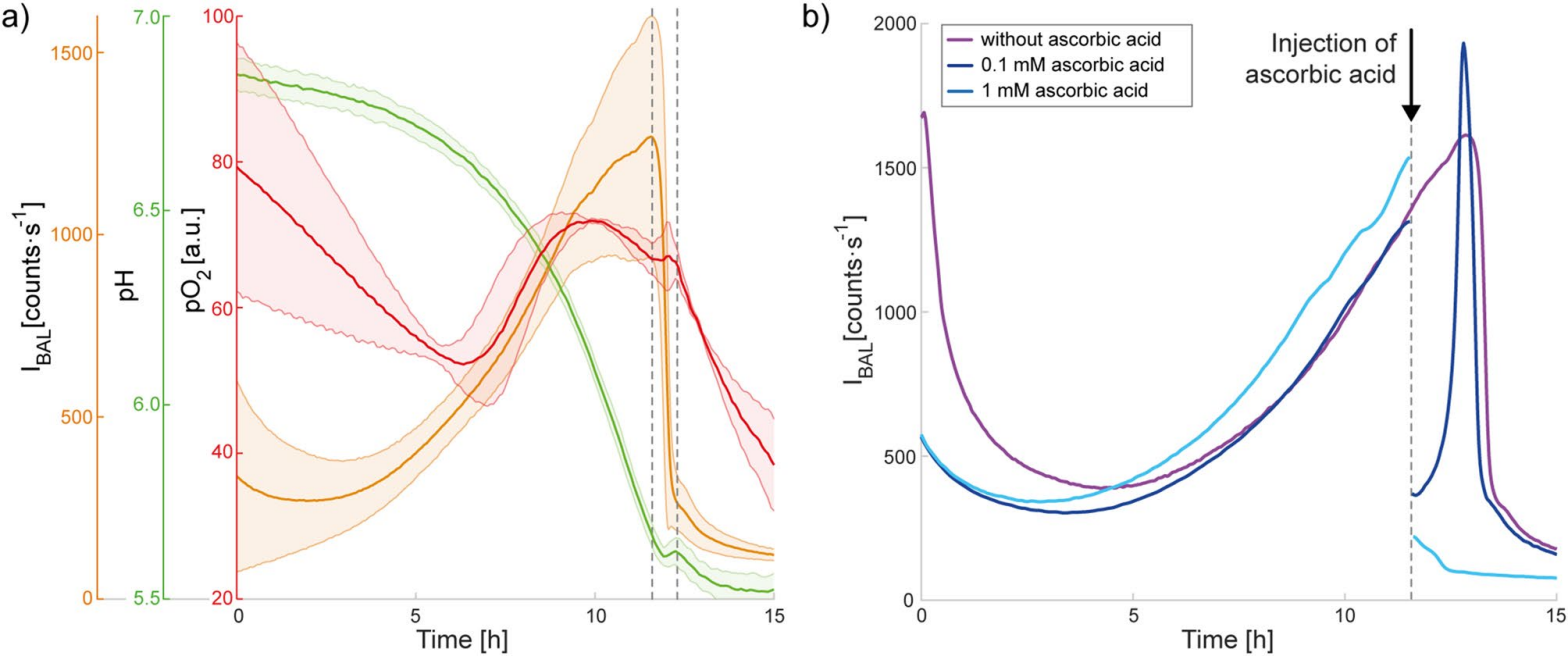
(**a**) Spontaneous BAL of yeast measured in a bioreactor together with the change in pH and ${\text {pO}}_2$ (average from 3 measurements with a standard deviation). Dashed vertical lines around the twelfth hour delimit the sharp decrease of the BAL intensity and corresponding changes in pH and ${\text {pO}}_2$. (**b**) Influence of various concentrations of the antioxidant ascorbic acid (0–1 mM) on spontaneous BAL of yeast. The initial concentration of the sample was $5 \cdot 10^5 \, \hbox {cells} \,\, {\hbox {mL}}^{-1}$.

We also analyzed the spontaneous BAL of yeast culture in a bioreactor during their growth (Fig. 4a). Because the sample was prepared under normal laboratory light conditions, we observed an initial slight decrease of the BAL signal while the sample was adapting to the dark condition in a black box (the decreasing signal was observed also in YPD medium only, see Fig. S2, so we do not attribute it to cellular processes). Then, the signal increased during several hours. At the same time, the pH decreased and ${\text {pO}}_2$ displayed nonlinear behavior. After approximately 12 h of measurement, the BAL signal sharply decreased, whereas the pH and ${\text {pO}}_2$ slightly increased. Quickenden et al. observed that the BAL of yeast *Saccharomyces cerevisiae* was similar to nutrient luminescence (i.e. close to background) in anaerobic conditions (nitrogen instead of air)^24^. However, there was still enough oxygen in our samples after approximately 12 h (Fig 4a). As shown in Fig. 3d, a transition from exponential to stationary phase of the cell growth curve occurs after approximately 12 h (in a control sample = without Fenton reagents treatment). This change of phases is called the diauxic shift and it is connected with a change in yeast metabolism from fermentation to aerobic respiration. Therefore, a sharp decrease of the BAL intensity could be caused by internal changes related with the initiation of respiration. This statement might look contradictory at first sight, because within classical understanding of physiological processes underlying BAL, a higher respiration rate (mitochondria-enabled) should lead to a higher rate of ROS generation^31–34^, hence to a higher BAL intensity. To explain our observations, we propose the following hypothesis:

1. the ROS generation rate $k_{\mathrm {ROS}}=\frac{d \mathrm {{ROS}}}{d\mathrm {t}}$ per unit of effective amount of metabolically active yeast biomass $M_{\mathrm {active}}$ during anaerobic phosphorylation is smaller than the one during the oxidative phosphorylation, i.e., $k_{\mathrm {ROS,anaerobic}} < k_{\mathrm {ROS,aerobic}}$
2. the metabolic activity of yeast is much higher during anaerobic metabolism than during aerobic metabolism, as demonstrated by the cell growth-rate in the exponential phase versus stationary phase, i.e., $M_{\mathrm {active,anaerobic}} \gg M_{\mathrm {active,aerobic}}$
3. the BAL intensity $I_{\mathrm {BAL}}$ is proportional to $M_{\mathrm {active}} k_{\mathrm {ROS}}$
4. $$M_{\mathrm {active,anaerobic}} \; k_{\mathrm {ROS,anaerobic}} > M_{\mathrm {active,aerobic}} \, k_{\mathrm {ROS,aerobic}}$$, i.e., $$I_{\mathrm {BAL, anaerobic}} > I_{\mathrm {BAL,aerobic}}$$

What could be the potential sources of ROS during anaerobic metabolism and is there any supporting data for our hypothesis? According to^35^, non-mitochondrial ROS generation can be related to cellular biosynthesis. Tilbury et al. also suggested that possible source of BAL during the exponential phase of yeast growth (during fermentation) are oxidative side-reactions accompanying protein synthesis^14^. Rinnerthaler et al. found that an important non-mitochondrial source of ROS in yeast is a NADPH-oxidase ortholog Yno1p/Aim14p^6^. Maslanka et al. also evaluated the content of pyridine nucleotide cofactors and the ratios of their reduced and oxidised form. They observed a higher ratio of ${\hbox {NADPH/NADP}}^{+}$ for yeast cultivated in the media supporting aerobic respiration^35^. Besides other functions, NADPH is considered to directly operate as an antioxidant to scavenge free radicals and reduce oxidised biomolecules^36^. Jamieson et al.^37^ observed significantly higher (oxidative) stress resistance during respiratory growth and stationary phase than during fermentation. Because incomplete reduction of ${\text {O}}_2$ during respiration is usually considered as the major source of ROS^5^, organisms have developed active, antioxidant-based defense mechanisms, which lead to a decreased amount of ROS in cells or repairing of the damage caused by them. Maslanka et al. observed a dependence of ROS amount and also the ROS source on the type of carbon source/concentration of glucose and on the type of metabolism of yeast *Saccharomyces cerevisiae* wild-type and two mutant strains: $\Delta$sod1 and $\Delta$sod2^35^. Their results showed that non-mitochondrial ROS sources are an important pool of ROS in yeast, especially during fermentation. They also observed that more ROS are generated by yeast in the medium with higher concentration of glucose when they estimated the level of ROS with dihydroethidium (DHET). Because glucose is an important source of energy for yeast and its concentration in a sample has influence on yeast metabolism, we also performed experiments with glucose. We observed changes in the glucose concentration in the yeast samples during their growth (Fig. S4) and an increase in the yeast BAL intensity when glucose was re-supplied into the sample (Fig. S3). Our data are in agreement with the results of Maslanka et al.^35^. The glucose concentration decreased with increasing yeast concentration. In our second mentioned type of the experiment with glucose, after the BAL signal reaches the peak and drops to a low level, the addition of glucose causes a temporary increase of the BAL intensity (Fig. S3).

To briefly comment on the kinetics of the pH and partial pressure of oxygen (${\text {pO}}_2$) in the sample during 15-h yeast growth in the bioreactor (Fig. 4a): The decrease of pH during fermentation is a well-known effect in yeast biotechnology caused by excretion of organic acids and absorption of basic amino acids^38^. The kinetics of ${\text {pO}}_2$ is much more enigmatic: Initially, there is a decrease of ${\text {pO}}_2$, then an increase starting after approximately 6–7 h and then a decrease again after 10 h. In general, there are two competing processes that determine the ${\text {pO}}_2$: ${\text {O}}_2$ (air) supply to the bioreactor via an injecting tube through a bubbling output and consumption of the ${\text {O}}_2$ in the sample, which explain the three observed phases of the ${\text {pO}}_2$ kinetics. The initial decrease of ${\text {pO}}_2$ might be due to slowly fading auto-oxidation taking place in the YPD medium, which binds ${\text {O}}_2$. The following increase might be due to ${\text {O}}_2$ supply starting to dominate over the ${\text {O}}_2$ consumption. The decrease that starts after 10 h is likely owing to the onset of cellular respiration.

We carried out further experiments to test whether oxidative reactions are indeed involved in spontaneous BAL generation. The amount of ROS in a sample can be increased by the Fenton reaction, as demonstrated in previous studies^26–28^. On the other hand, the sample ROS content can also be artificially decreased by using, for example, an antioxidant. For experiments in which we wanted to suppress ROS (with an expected consequence of suppressing the BAL intensity), a sample with a higher BAL intensity is more suitable for observing a significant signal decrease. Therefore, the influence of the antioxidant ascorbic acid on the BAL of yeast culture was observed at the time points with high BAL intensity (Fig. 4b). The effect of the antioxidant depended on its final concentration in the sample. After injection of ascorbic acid at a lower concentration (0.1 mM), we observed a rapid decrease followed by a steep increase back to the values expected for a sample without antioxidant treatment. The injection of ascorbic acid at a higher final concentration (1 mM) resulted in a steep decrease and no renewal of the signal. The observed effects probably depended on the ratio of the amounts of ROS and antioxidant. At a lower final concentrations of antioxidant, the molecules of ascorbic acid react with ROS and are quickly depleted. Then, the amount of ROS, and thus the BAL signal, increases again without any hindrance. A higher concentration of ascorbic acid either terminates the chain production of ROS or considerably reduces the amount of ROS for such a long time that the yeast can pass through the fermentation to the aerobic respiration phase. Červinková et al.^39^ also studied the effects of ascorbic acid (vitamin C), among other antioxidants, on yeast BAL intensity. They tested concentrations of 1, 5, and 10 mM and also did not observe any renewal of the BAL signal. They also tested the influence of ascorbic acid (1, 500, and $1000 \, \upmu \hbox {M}$) on the BAL intensity of HL-60 cells. Even at the lowest used concentration ($1 \, \upmu \hbox {M}$), the signal did not recover. As expected, each sample has a different threshold antioxidant concentration and also a different response to various antioxidants^39^.

## Conclusion

We investigated the correlations between selected physical, chemical, and biological factors and the BAL intensity of yeast samples. The oxidation of a yeast cells using the Fenton reaction caused a higher BAL signal with increasing concentrations of the Fenton reagents. A higher BAL signal was also observed with increasing yeast concentration up to a certain limit, after which the BAL signal decreased again. On the other hand, the addition of the antioxidant ascorbic acid to the sample decreased the BAL intensity for some time depending on the antioxidant dose. We did not observe any damaged yeast cells under a microscope after short-time oxidation, just the tendency of the cells to cluster. However, a higher ${\text {H}}_2 {\text {O}}_2$ concentration caused prolongation of the lag phase of the yeast growth.

During yeast culture growth in a bioreactor, the BAL intensity increased together with the number of cells, the pH and glucose concentration decreased, whereas the change in ${\text {pO}}_2$ was nonlinear. After approximately 12 h, when the glucose concentration was low and the yeast samples were undergoing the growth phase change (diauxic shift), the BAL signal sharply decreased.

We demonstrated that the BAL intensity of the yeast sample is proportional to the amount of ROS in the sample. Our data indicated that non-mitochondrial sources of ROS also play an important role in the production of ROS and BAL. We were able to monitor various processes closely connected with the change of ROS content in the studied object by observing changes of the BAL intensity over time. However, the ROS content can be affected by many various factors. Beyond chemical factors, it could be also physical factors such as magnetic^29^ and electric field, particularly pulsed electric field, which is a part of our ongoing work. In general, knowing which other factors can influence the BAL intensity and the mechanisms involved will allow the development of a new reliable diagnostic method.

## Methods

Yeast *Saccharomyces cerevisiae* wild type (BY 4741) was used as a model organism. They were cultivated for approximately 24 h in an orbital shaking incubator (Yihder Co., Ltd; LM-420D) at $30\,^\circ \hbox {C}$, 180 rpm, in standard YPD medium (1 % yeast extract, 2 % peptone, 2 % D-glucose). Then, they were centrifuged (Heraeus Biofuge Stratos) 2 times and the YPD medium was changed to Milli-Q ${\text {H}}_2 \hbox {O}$. Measurement of yeast concentration was performed with a cell counter (Beckman Coulter, Z2 series) in the range $3 \, \hbox {-} \, 9\, \upmu \hbox {m}$. Yeast *Saccharomyces cerevisiae* has a diameter between 1 and $10\, {\upmu } \hbox {m}$. Their mean cell size depends on various factors such as growth temperature^40^, strain, and age^41^.

### Biological autoluminescence measurement setup

#### Induced BAL

Induced emission was measured from a 3 mL of yeast sample in Milli-Q water in a Petri dish (Thermo Scientific, diameter 35 mm). The concentration of yeast was between $10^7$ and $10^9 \, \hbox {cells} \,\, {\hbox {mL}}^{-1}$. The amount of ROS in the sample was regulated by the Fenton reaction

1 $$\begin{aligned} \mathrm {Fe^{2+} + H_2O_2 \rightarrow Fe^{3+} + HO^{\cdot } + OH^-} \end{aligned}$$

**Figure 5:**
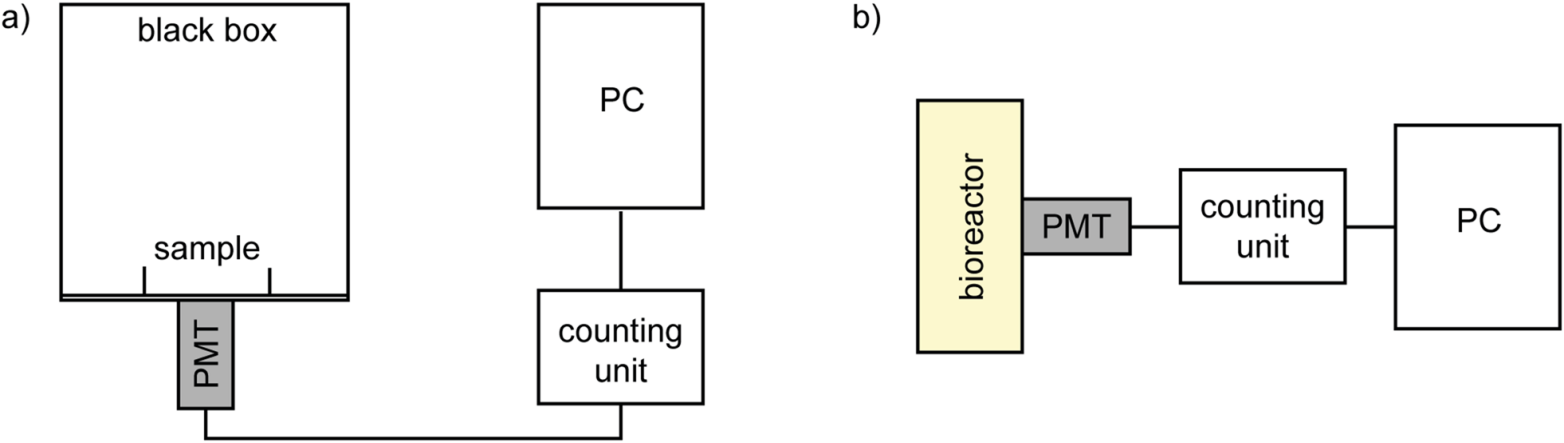
A scheme of the experimental setup for the measurements of BAL during (**a**) induced oxidation in a Petri dish in a black box and (**b**) spontaneous oxidation in a bioreactor. The bioreactor with the photodetector chamber was light-isolated by a black cloth construction.

After preparation of a yeast sample at a required cell concentration, $50\, \upmu \hbox {L}$ of iron(II) sulfate was added to the sample so that the final ${\text {FeSO}}_4$ concentrations in the sample was 50 nM, $5\, \upmu \hbox {M}$, or 0.5 mM. Then the sample was placed into a special black box with a photomultiplier module H7360-01 (Hamamatsu Photonics K.K., spectral range 300–650 nm) where the measurements of BAL were performed. After approximately 1 min of BAL measurement, $50\, \upmu \hbox {L}$ of ${\text {H}}_2 {\text {O}}_2$ (final concentrations: $25\, \upmu \hbox {M}$, $250\, \upmu \hbox {M}$, 2.5 mM, and 25 mM) was injected into the yeast sample with ${\text {FeSO}}_4$. The whole BAL measurement took approximately 10 minutes. A scheme of the experimental setup is shown in Fig. 5a.

#### Spontaneous BAL

Spontaneous emission was measured in a bioreactor Biostat Aplus (Sartorius Stedim Biotech) in a dark room. The bioreactor with the photodetector chamber was light-isolated by a black cloth construction to further minimize any background light. Yeasts at an initial concentration of $5 \cdot 10^5 \, \hbox {cells} \,\, {\hbox {mL}}^{-1}$ were stirred (180 rpm), bubbled with air (${\sim } 1 \, \hbox {L} \,\, {\hbox {min}}^{-1}$) and maintained at a temperature of $30\,^\circ \hbox {C}$ during their cultivation in YPD medium. $75\, {\upmu } \hbox {L}$ of antifoaming agent (polypropylene glycol) was added to the 750 mL sample. The measurement time was between 15 and 20 h. The photomultiplier module (H7360-01, Hamamatsu Photonics K.K., spectral range 300–650 nm) was used for BAL measurements. A scheme of the experimental setup is shown in Fig. 5b. Measurement of the pH, ${\text {pO}}_2$, and temperature was performed by original probes included in the bioreactor.

The initial yeast concentration is lower compared with measurements of BAL intensity after short-time oxidation of yeast cells in water induced by the Fenton reaction. If we want to measure spontaneous BAL of yeast and their growth curve simultaneously, it is better to start at a lower yeast concentration to have a nicer shape of the curve. (The higher initial amount of yeast quickly depletes the glucose , the exponential phase is really short, and the difference between the initial and the final cell concentration is not so distinct.) Also, the measurement of yeast concentration is easier and more precise (without dilution of a sample) in the used concentration range. (Upper limit of a sample concentration is approximately $3 \cdot 10^8 \, \hbox {cells} \,\, {\hbox {mL}}^{-1}$ for the cell counter.)

The experiment for observing the influence of glucose re-supply into the sample on BAL intensity was performed in an Erlenmeyer flask in a black box similar to the measurements of induced BAL. The measurement setup is described in^42^. Cells were cultivated in an orbital shaking incubator (YPD, $30\,^\circ \hbox {C}$, 180 rpm) for 16 h. The cell concentration in the sample was established with a Bürker chamber. The required amount of yeast cells was transported to 200 mL of cold YPD medium so that the initial yeast concentration was $5 \cdot 10^5 \, \hbox {cells} \,\, {\hbox {mL}}^{-1}$. The sample was bubbled with filtered room air. After 16 h of spontaneous BAL measurement, 11 mL 40% glucose was added into the sample.

### Glucose concentration measurement

The glucose concentration was established with a commercial glucose kit (Glu 1000, Erba Lachema). Optical density was measured at 500 nm and subsequently recalculated to a real glucose concentration in mM (using calibration solutions at known glucose concentration).

### Growth curve

Growth curves were measured in a Spark microplate reader (Tecan). After approximately 24-h cultivation of yeast in an orbital shaking incubator ($30\,^\circ \hbox {C}$, 180 rpm) in standard YPD medium, the samples ware centrifuged (2$\times$) and the YPD medium was changed to Milli-Q ${\text {H}}_2 \hbox {O}$. Then, oxidation of the yeast sample (concentration of $10^8 \, \hbox {cells} \,\, {\hbox {mL}}^{-1}$) was performed with the Fenton reaction ($0.5\, \hbox {mM} \, {\text {FeSO}}_4$, 2.5 mM/25 mM/250 mM ${\text {H}}_2 {\text {O}}_2$) for 10 min. The samples were in the shaker ($30\,^\circ \hbox {C}$, 180 rpm) during the oxidation. Then, the samples were centrifuged and the water was changed to fresh YPD medium. After dilution of each sample 100 times, $200\, {\upmu } \hbox {L}$ of each sample was put in a well of a 96-well plate (Thermo Scientific Nunc, transparent, non-treated, flat bottom, type no. 269620) and covered with a lid. 5–7 replicates of each sample were prepared on the same plate. The optical density at 600 nm was measured every 10 min for 24 h. The samples were shaken for the rest of the time. The temperature during measurement of the growth curves was maintained at $30\,^\circ \hbox {C}$.

### Microscopic imaging

Microscopic imaging before and after the oxidation by the Fenton reaction was performed with the microscope BX50 (Olympus) with the camera Moticam 1080. Phase contrast and an objective magnification of 20$\times$ was used.

## Supplementary Information

Supplementary Information 1.

## Data Availability

Raw data are available under 10.5281/zenodo.4382342 and described in the supplementary information S1.
